# Assessment of the Genetic Diversity and Structure of the Korean Endemic Freshwater Fish *Microphysogobio longidorsalis* (Gobioninae) Using Microsatellite Markers: A First Glance from Population Genetics

**DOI:** 10.3390/genes15010069

**Published:** 2024-01-04

**Authors:** Kang-Rae Kim, Mu-Sung Sung, Yujin Hwang, Ju Hui Jeong, Jeong-Nam Yu

**Affiliations:** 1 Animal & Plant Research Department, Nakdonggang National Institute of Biological Resources, Sangju 37242, Republic of Korea; kimkangrae9586@gmail.com (K.-R.K.); dbwls8@nnibr.re.kr (Y.H.); juhii22@nnibr.re.kr (J.H.J.); 2 Muldeuli Research, Icheon 12607, Republic of Korea; rkrtlqndj@naver.com

**Keywords:** microsatellite, bottleneck, population structure, genetic diversity, endemic, *Microphysogobio longidorsalis*

## Abstract

*Microphysogobio longidorsalis* is endemic to South Korea and inhabits small areas of the Namhangang, Bukhangang, and Imjingang Rivers in the Hangang River water system. Endemic species usually are more vulnerable than species with a wide distribution. Notably, there is a lack of basic conservation data for *M. longidorsalis*. We analyzed 19 microsatellite loci in six populations of *M. longidorsalis* in South Korea to characterize their population structure and genetic diversity. The genetic diversity of the microsatellites was 0.741–0.779, which is lower than that of other freshwater fishes. The pairwise genetic differentiation of microsatellite (*F*_ST_) values ranged from 0.007 to 0.041, suggesting low genetic differentiation between the populations. The Jojongicheon stream population (CP) had an effective population size of <100. Therefore, conservation efforts are required to prevent inbreeding depression in *M. longidorsalis*. Discriminant analysis of principal components showed that the Hangang River water system would be a single management unit (MU). Our findings provide fundamental genetic insights for the formulation of conservation strategies for *M. longidorsalis*.

## 1. Introduction

**Figure 1:**
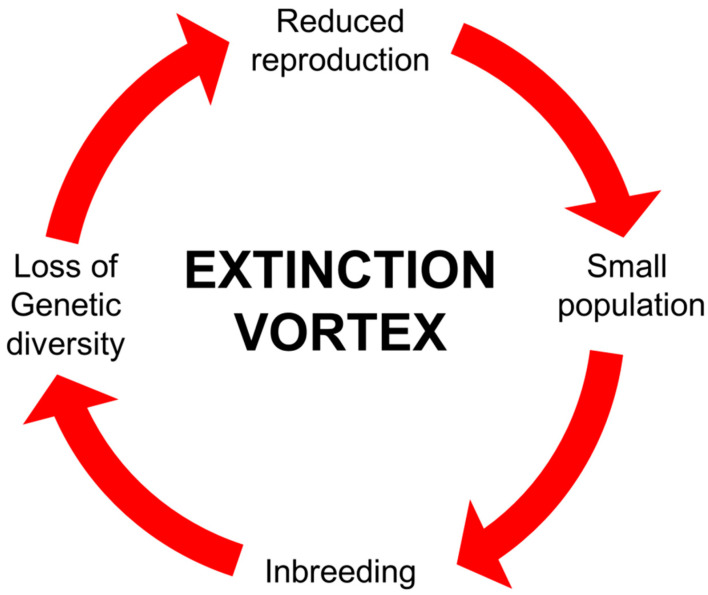
Overview of the extinction vortex.

The protection of endemic species that are confined to specific habitats is difficult and complex in conservation biology [1]. Threats can significantly affect individuals or populations owing to their confined area distribution [2]. Species with small distributions are vulnerable to persistence (vulnerable to extinction) as they have fewer individuals and populations than those with wide distributions [1,2]. The perception that most well-known or economically valuable species require conservation efforts [3] results in most species being excluded from conservation efforts despite the need for their inclusion. Therefore, no conservation efforts have been made to reintroduce or increase the population of endemic species outside of concern [2]. Endemic species with small distribution ranges are thought to undergo allele loss owing to genetic drift and low genetic diversity [4,5]. Notably, deleterious alleles are likely to occur at a higher frequency in smaller populations than larger populations owing to their small effective population sizes [6]. Subsequently, their physical fitness generally decreases, they become prone to extinction, and their reduced genetic diversity makes them less capable of coping with environmental changes than larger populations [6]. These threats increase the likelihood that they will fall into an extinction vortex and become endangered (Figure 1) [7].

We are currently experiencing the sixth mass extinction [8]. Vulnerability to extinction can be attributed to five factors:

1. Excessive exploitation by humans [9];
2. A decrease in population size [10];
3. Low reproductive potential [11];
4. Habitat damage due to human activities [12];
5. The threat of extinction due to invasive species [13].

More than half of these five factors are likely caused by human activity. This may include reduced physical fitness owing to the confined area distribution of endemic species and increased threat from habitat damage caused by human activities. Species affected by these factors require effective conservation strategies and should be prioritized for conservation [5].

Species on the Korean Peninsula used to be connected to each other by rivers, but as mountain ranges rise, genetic exchange is hindered, resulting in genetic differences [14]. Recognizing genetic differences and structures is important for conservation planning [4]. Genetic diversity is an essential component of conservation and management in conservation biology [2]. Endemic species sometimes have low genetic diversity, which leads to a decrease in population size due to habitat damage from anthropogenic activities, ultimately resulting in lower genetic diversity [1,4,15]. The Bukhangang, Namhangang, and Imjingang Rivers are connected to each other at a brackish water area where seawater and freshwater mix. However, whether genetic exchange is actually occurring in *M. longidorsalis* has not been studied. In this study, the PC and JC sampled in the Namhan River are blocked by the Chungju Dam. Therefore, there is a need to analyze whether the impact of the dam will lead to the formation of different populations due to the disconnection. The CP, GP, and HC of the Bukhangang River were sampled below the Hwacheon Dam and Soyang River Dam. The Bukhan River was sampled downstream of the dam because the distribution of *M. longidorsalis* could not be found upstream. The sampled areas are generally urbanized areas around rivers, into which sewage pollution flows.

In the genus *Microphysogobio*, *M. rapidus* and *M. koreensis* are endangered species [16]. In particular, *M. rapidus* is a critically endangered (CR) species on the IUCN Red List and is close to extinction. (https://www.iucnredlist.org/search?query=Microphysogobio%20rapidus&searchType=species, accessed on 15 November 2023). The *Microphysogobio* genus primarily inhabits habitats with rapids [16]. Threats, such as habitat destruction and the construction of dikes, dams, and river embankments, have made them vulnerable to extinction. *Microphysogobio rapidus* previously inhabited over 10 locations in the Nakdonggang River water system alone. Currently, it inhabits only two locations and is on the verge of extinction due to habitat destruction resulting from human activities [17,18]. *M. longidorsalis*, like *M. rapidus*, belongs to the genus *Microphysogobio* and only inhabits a limited area, that is, the Hangang River basin in South Korea [16]. This limited distribution could lead to extinction for this species, especially in rivers in urbanized areas (Bukhangang River).

*Microphysogobio longidorsalis* lives on rapid and gravelly bottoms [16]. It also inhabits the Hangang and Daedonggang River water systems [16]. In South Korea, it is found only in the Hangang River and has a small distribution range [16]. To date, no genetic or habitat studies have been conducted on *M. longidorsalis* conservation. As in the case of *M. rapidus*, population size reduction and habitat destruction due to sewage pollution or river construction can have fatal consequences, threatening extinction [17]. Therefore, *M. longidorsalis* is currently experiencing a decline in its habitat (Chungju Dam, Hwacheon Dam, and Soyang River Dam), and because it lacks basic conservation data and is distributed in a limited area, there may be a threat of extinction. *M. longidorsalis* lives in the Han River water system, which is divided into the Namhangang, Bukhangang, and Imjingang rivers. Understanding genetic structure is one of the foundations of conservation [1]. Management units (MUs) are a key element in managing wild populations. It has been suggested that the identification of MUs should be based on the amount of genetic divergence [18]. The genetic structure of *Pseudopungtungia tenuicorpa* (a species belonging to the Gobioninae family that lives in the Hangang River) is divided between the Bukhangang and Namhangang rivers [19]. Therefore, the genetic structure of *M. longidorsalis* may also be divided among the Namhangang, Bukhangang, and Imjingang rivers. Microsatellites have been mainly reported in studies on the genetic structure and diversity of the Gobioninae subfamily. Consequently, significant genetic diversity and structures were reported [20,21,22]. Microsatellites are commonly analyzed in population genetics studies because of their high intraspecific polymorphism and codominance [23,24]. Population genetics investigations, such as microsatellite analyses of genetic diversity, have not yet been performed for *M. longidorsalis*.

In this study, we analyzed the genetic diversity of *M. longidorsalis* using microsatellite markers at six sites in the Hangang River water system: two sites in the Namhangang River, three in the Bukhangang River, and one in the Imjingang River. The genetic structure was identified to provide basic data for establishing conservation strategies.

## 2. Materials and Methods

### 2.1. Sampling and DNA Extraction

**Figure 2:**
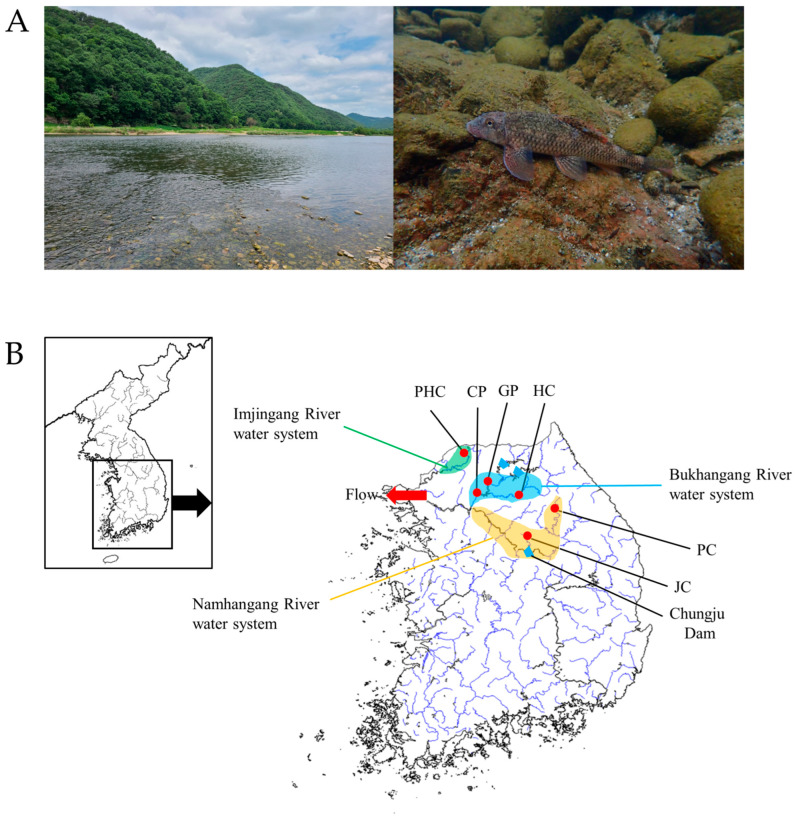
(**A**) Photo of *M. longidorsalis* and its habitat. (**B**) Sampling sites of *M. longidorsalis* and distribution of sampling sites. Yellow: Namhangang River water system; blue: Bukhangang River water system; green: Imjingang River water system. The abbreviations for the populations are presented in Table S1. PC: Hongjeongcheon stream; JC: Jecheon stream; GP: Gapyeongcheon stream; HC: Hongcheon River; CP: Jojongicheon stream; PHC: Yeongpyeongcheon stream.

*M. longidorsalis* is endemic to Korea; therefore, the need for animal ethics approval was waived. Six *M. longidorsalis* populations were sampled using fishing nets at points representative of the genetic diversity of the populations (details provided in Figure 2 and Table S1). Because it was expected that there would be genetic differences in the Namhangang, Bukhangang, and Imjingang Rivers, we sampled two locations upstream of the Namhangang River, three locations upstream of the Bukhangang River, and one location upstream of the Imjingang River. A total of 120 specimens were collected, with 20 specimens from each population. The collected samples were stored by soaking the whole body of *M. longidorsalis* in 99.9% alcohol. Sampling was conducted between February and May 2023, and fin tissue from the fish was cut (10 mg) and soaked in 99.9% ethanol for genomic DNA extraction. Genomic DNA was extracted using a DNeasy Blood & Tissue Kit (QIAGEN, Hilden, Germany) according to the manufacturer’s instructions. The extracted genomic DNA was stored at −20 °C after dilution to 50 ng/μL to amplify the microsatellite region.

### 2.2. Whole-Genome Sequencing for Microsatellite Screening

HiFi and Illumina sequencing were performed on one specimen from the Bukhangang River. We employed Pacific BioSciences (PacBio, Menlo Park, CA, USA) HiFi single-molecule real-time (SMRT) sequencing following the manufacturer’s protocol (Macrogen Inc.). The HiFi SMRT cell library was prepared using the SMRTBell Express Template Prep Kit 2.0 (PacBio). The gDNA was cut into 6–20 kb fragments using a g-TUBE (Covaris, Woburn, MA, USA), and the reagents included in the Template Prep Kit were used. Notably, this also repairs damaged DNA and fragment ends. A SMRTbell hairpin adapter was attached to the repaired extremity. AMPure PB beads (PacBio) were used for library enrichment and purification. SMRTbell template sizes of >10 kb were selected using the BluePippin system (Sage Science, Beverly, MA, USA). HiFi reads generated from the PacBio SequelII system were assembled using the genome assembly application SMRT link v. 11.0.0.146107 (NovogeneAIT, Singapore). All the options used in the genome assembly analysis were set to their default values. Short-read sequencing was performed to determine the reliability of the base sequences of the long reads. The whole genome was sequenced using a 150 bp paired-end library produced by Macrogen (Macrogen Inc., Seoul, Republic of Korea) on an Illumina HiSeq 2500 platform (Illumina, San Diego, CA, USA). Illumina short-read Trimmomatic ver. 0.33 [25] clean reads were obtained after removing the adapter sequences. BWA-MEM [26] and Pilon version 1.23 [27] were used to improve the accuracy of the initial contig assembly. Contigs with more than five repetitions were selected for di-, tri-, tetra-, penta-, and hexa-sequences using the MISA tool (http://misaweb.ipk-gatersleben.de/, accessed on 15 October 2023). Primer3 (https://github.com/primer3-org/primer3, accessed on 15 October 2023) was used to set the length of the appropriate primer to 20–24 bp, the size of the amplification product to 150–400 bp, and the melting temperature (Tm) to 58–60 °C.

### 2.3. Microsatellite Genotyping

One hundred microsatellite loci were randomly selected. PCR was performed using a Mastercycler^®^ pro gene amplifier using 10 ng genomic DNA, 0.5 units H-Taq polymerase (BioFact, Daejeon, Republic of Korea), 0.4 μM forward primer, 0.8 μM reverse primer, and 0.4 μM labeled fluorescent label (FAM, VIC, NED, and PET). The final volume of the reaction mixture was 20 μL, including the M13 (TGTAAAACGACGGCCAGT) primer labeled with fluorescent dyes FAM, NED, VIC, and PET. The PCR cycling conditions included pre-denaturation at 94 °C for 5 min, followed by 30 cycles of denaturation at 94 °C for 30 s, annealing at 58 °C for 45 s, and extension at 72 °C for 45 s, according to the method described by Schuelke [28]. This was followed by eight cycles of denaturation at 94 °C for 30 s, annealing at 53 °C for 45 s, and extension at 72 °C for 45 s. The final extension was performed at 72 °C for 10 min, and the temperature was maintained at 4 °C. The amplified PCR products were electrophoresed on 1.5% agarose gel to confirm the presence or absence of bands and size of the amplified fragment. Microsatellite PCR product fragments were prepared by mixing a GeneScan™ 500 ROX size standard ladder (Applied Biosystems, Foster City, CA, USA) and HiDi™ formamide (Applied Biosystems), and denaturation was performed at 95 °C for 2 min, followed by termination at 4 °C. Allele sizes were determined using an ABI 3730xl DNA Analyzer (Applied Biosystems). Genotyping was performed using the GeneMarker^®^ 2.6.7 program (SoftGenetics, State College, PA, USA). Microsatellite markers were deposited in GenBank under accession numbers OR722786–OR722804.

### 2.4. Microsatellite Genetic Diversity Analyses

MICROCHECKER software (ver. 2.2.3) [29] was used to examine the presence or absence of scoring errors at the microsatellite loci. Genetic diversity was measured as the number of alleles (*N*_A_), expected heterozygosity (*H*_E_), and observed heterozygosity (*H*_O_) using CERVUS software (ver. 3.0) [30]. Population inbreeding coefficient (*F*_IS_) and Hardy–Weinberg equilibrium (HWE) variance analyses were performed using GENEPOP (ver. 4.2) [31] and ARLEQUIN software (ver. 3.5) [32]. Two methods were used to estimate the bottlenecks. These methods used BOTTLENECK software (ver. 1.2.02) [33], a program to estimate bottlenecks through excess heterozygosity testing, and an infinite allele model (IAM) [34]. A two-phase model (TPM) and stepwise mutation model (SMM) [35] were used for the estimation, and the TPM was performed with 10% variance and 90% SMM. In addition, each model had 10,000 iterations, and the significance was verified using the Wilcoxon signed-rank test [36]. Contemporary effective population size (Ne) was estimated for each population using NeEstimator ver. 2.1 software [37] with the Linkage Disequilibrium (LDNe) method under a random mating model [37].

### 2.5. Population Genetic Structure Analysis

ARLEQUIN software (ver. 3.5) [32] was used to analyze the genetic differences between the groups and molecular variance (AMOVA). AMOVA was performed with permutations of 1000. AMOVA was divided into two groups (Namhangang River: JC, PC vs. Bukhangang and Imjingang Rivers: GP, HC, CP, PHC). STRUCTURE software (ver. 2.3.4) [38] was used to perform genetic structure clustering analysis based on the Bayesian model. We set the population constant (*K*) to 1–10, and applied a suitable admixture model to a mixture of water systems to estimate the most suitable population. Ten independent replicates with a burn-in of 10,000 iterations and Markov chain Monte Carlo (MCMC) with 100,000 iterations were performed. Two K estimators were used to identify the most likely number of clusters: the Mean LnP(K) [38] and the deltaK ad hoc estimator [39]. We analyzed a study by Evanno et al. [39] and the cluster results corresponding to *K* using STRUCTURE HARVESTER (https://taylor0.biology.ucla.edu/structureHarvester/, accessed on 10 October 2023) [40] to estimate a population-appropriate constant (*K*). A discriminant analysis of principal components (DAPC) was performed using the R ADEGENET (ver. 2.1.3) package [41], a non-model-based genetic clustering method. The “find.clusters” function belonging to the R package ADEGENET applied clusters to the data according to BIC (Bayesian Information Criterion) values.

## 3. Results

### 3.1. Microsatellite Genetic Diversity

In total, 5,468,650 Hi-Fi reads were obtained using whole-genome sequencing. The total read length was 33,151,752,272 bp. The number of short reads was 725,452,696, and the length was 109,120,602,840 bp. The number of assembled contigs was 6220, which were used to screen the microsatellite region. The assembly and microsatellite screening results are presented in Table S2. A total of 100 markers were screened. Markers that deviated from HWE were excluded, and 19 markers were used for population genetic analysis. Each of the 19 microsatellite markers was evaluated in 120 individuals from the six populations. Genotypes were analyzed in all individuals, with a missing rate of 0. The polymorphic information content (PIC) values of the 19 selected microsatellite loci varied from 0.253 to 0.938; the 19 selected markers had high values (0.253–0.938), representing genetic diversity, and were used in the population genetics analyses. The number of alleles ranged from 3 to 22. HWE analysis did not yield significant results for any of the 19 loci; therefore, they were used for population analysis. Genotyping using MICRO-CHECKER software (ver. 2.2.3) showed no evidence of scoring errors or null alleles (Table S3).

Nineteen microsatellite loci were analyzed for the genetic diversity indices of the six populations (Table 1). The number of alleles, *H*_O,_ and expected heterozygosity ranged from 7.74 to 9.47, 0.625 to 0.683, and 0.741 to 0.779, respectively. All populations deviated from HWE. The inbreeding index was above 0.079 in all populations, and the *F*_IS_ was significant (*p <* 0.05). The observed heterozygosity was highest in the PC population (*H*_O_ = 0.683) and lowest in the JC population (*H*_O_ = 0.625); however, the observed heterozygosity of all populations was not significantly different.

### 3.2. Bottleneck Analysis

We identified significant bottlenecks in all populations (*p* < 0.05) using the IAM. The TPM identified a bottleneck in the CP and PHC populations (Table 2). The CP populations exhibited recent mode shifts, demonstrating evidence of bottlenecks. 

The effective population size ranged from 78 to 688 among the six populations, and the HC and PHC populations appeared to be infinite. The effective size of the CP population was 78, which was the smallest (Table 2). However, these results may be erroneous owing to the small sample size. The CP population had a minimum effective population size of 100, which is necessary to prevent inbreeding depression.

### 3.3. Population Structure and Genetic Differentiation Analyses

Most *F*_ST_ values in the microsatellite dataset were significant, with the highest *F*_ST_ value between GP and CP (*F*_ST_ = 0.041). The PHC group had lower *F*_ST_ values than those of the GP and HC groups (*F*_ST_ = 0.172). *M. longidorsalis* had low *F*_ST_ values, indicating low genetic differentiation (Table 3).

**Figure 3:**
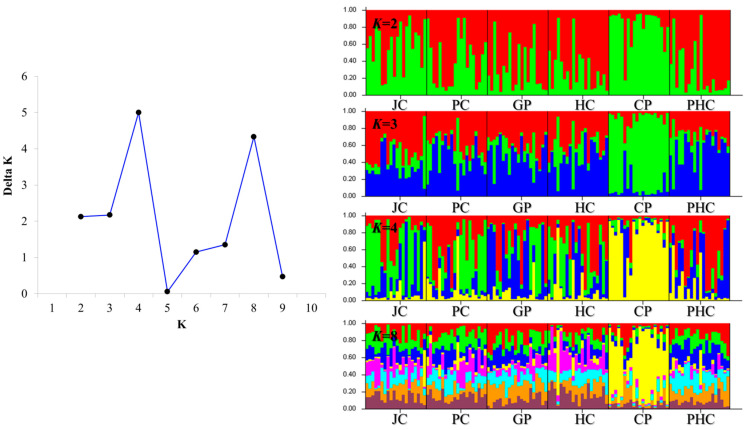
Population genetic structure of *M. longidorsalis*. Appropriate delta *K* information for the population constants. A single histogram represents the probability that an object and a particular color are assigned to a particular cluster.

**Figure 4:**
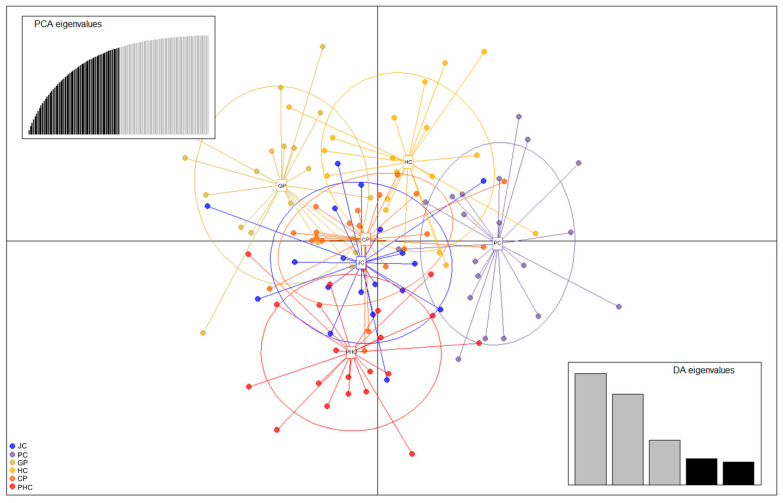
Scatterplots of discriminant analysis of principal components (DAPC). The numbers shown in the plot are the population IDs. Colored dots of different shapes represent individuals from different geographic populations, and the PCA and DA scatterplots on the right side of the graph represent the number of principal components and discriminant functions for calculation.

**Figure 5:**
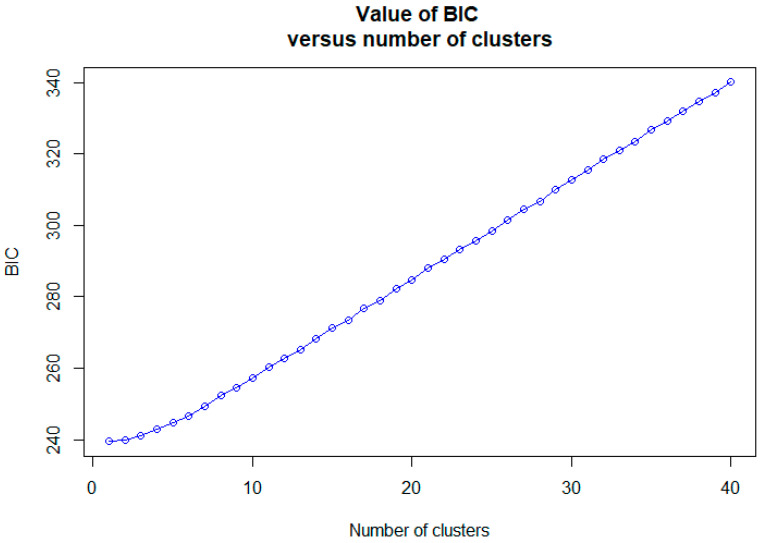
Value of BIC versus number of clusters. The BIC value closest to 0 indicates the best fit for the cluster.

Bayesian clustering analysis maximized the delta *K* values for the population structure at *K* = 4 and 8 (Figure 3). Significant genetic mixing was observed between the populations at *K* = 4. At *K* = 8, significant genetic mixing was observed between populations, with *K* = 4. The results of DAPC, which analyzes the structure of a population based on a non-model methods, showed that each population (JC, PC, GP, HC, CP, and PHC) was mixed, indicating that it was one population, unlike the STRUCTURE analysis results (Figure 3 and Figure 4). Therefore, we measured the value of LnP (K). It was found to be closest to 0 at −8928.47 at *K* = 1 (Table 4). Because the structure software uses a model approach, in this figure, *K* = 1 has a delta *K* value of 0. This is not possible because there is no delta *K* value for *K* = 1. The best fit was found to be at *K* = 1 because the value of LnP (K) was closest to 0. The ‘find.cluster’ function, implemented in the software ADEGENET (ver. 2.1.3), using all PCs, was applied to determine the best-supported number of genetic clusters using the Bayesian information criterion (BIC). The maximum number of clusters assayed was double that of populations, at 100,000 iterations per run, and 110 starting centroids were used. BIC was closest to 0 at K = 1, indicating the best fit at K = 1. This suggests that it is a single population (Figure 5).

The AMOVA for *M. longidorsalis* was performed on six populations to determine the genetic structure (Table 5). The AMOVA was 2.23% based on microsatellites among the populations within the groups, and 0.10% among the groups. AMOVA revealed a within-population variance of 97.66%.

## 4. Discussion

### 4.1. Genetic Diversity and Population Bottleneck

Genetic diversity aids in species adaptation and their ability to cope with the environment, and plays a key role in the persistence of species during evolution [1]. This study observed a relatively medium level of microsatellite genetic diversity in *M. longidorsalis*, ranging from 0.625 to 0.683, compared to *M. rapidus* (*H*_O_ = 0.757 [21]). Fish belonging to the genus *Microphysogobio* exhibit relatively high genetic diversity [21]. *M. rapidus* only exists in the Nakdonggang River water system [17]. *M. longidorsalis* exists in the Hangang and Daedonggang River water systems; however, it only exists in the Hangang River water system in South Korea. Despite its high genetic diversity, *M. rapidus* is on the verge of extinction due to habitat destruction and pollution. Considering the relatively low genetic diversity of *M. longidorsalis*, it may be more vulnerable to extinction if its population declines due to habitat destruction or pollution, as is the case with *M. rapidus*. *M. longidorsalis* has a confined area distribution because it only lives in the Hangang River basin in the Republic of Korea, and this small distribution poses relatively high threats such as anthropogenic habitat destruction. Therefore, strategies for coping with environmental changes by conserving genetic diversity should be considered in the future.

A few loci were strictly followed in the SMM, and the excess heterozygosity estimation method expected in the IAM was suitable for estimating recent bottlenecks [31]. The IAM results revealed that all populations were recently bottlenecked (*p* < 0.05). Additionally, the CP and PHC populations showed significant values using the TPM (*p* < 0.05). Therefore, a bottleneck phenomenon has recently occurred in all populations; in particular, a shifted mode in CP strongly supports the occurrence of a bottleneck phenomenon. Human activities such as habitat destruction and pollution reduce population size and create bottlenecks [1]. Previously encountered bottlenecks may have occurred because of human activities and simple sampling bias due to the small sample size [40]. A similar case has been reported by Kim et al. [42]. Additional research with a larger population size is necessary. However, in this study, the analysis was inevitably performed with 20 populations, because *M. longidorsalis* is a relatively difficult species to catch. Nonetheless, the six populations in this study appeared to experience bottlenecks. Therefore, conservation efforts are needed to establish legal conservation areas that can prevent the construction of weirs and dams.

Inbreeding often reduces the survival and reproduction of specific populations [19] and increases homozygosity, ultimately resulting in a loss of genetic diversity [1,43]. In all populations, HWE escaped, and the inbreeding coefficient (*F*_IS_) was significant (*p* < 0.001), showing relatively high inbreeding within *M. rapidus* (*M. longidorsalis*, *F*_IS_: 0.079–0.132; *M. rapidus*, *F*_IS_: 0.000–0.056). This suggests that inbreeding occurred in all populations. Inbred species experience suppression and reduced evolutionary capacity. Inbreeding has a negative effect on the persistence of a species, although this effect is less pronounced in large populations [1,43].

*N*e represents the ability to adapt to changing environments [1]. Small population sizes can accelerate local extinction risk owing to genetic drift and inbreeding depression effects [4]. This study measured Ne for the CP and GP populations; however, it could not be measured for the JC, PC, HC, and PHC populations. This is because the population size is small, and the estimate is negative; therefore, it is necessary to sample a larger population. However, because this species is currently difficult to catch due to pollution, it is important to measure the effective population size by increasing the population size in the future. The effective population size of the CP population was significantly smaller (Ne < 100). In contrast, the GP population had a large effective population size, with an Ne value of 1110. Frankham et al. [7] suggested that Ne should be >100 to avoid inbreeding depression. However, the CP populations have Ne values <100, indicating that they are likely to suffer from inbreeding depression. The CP population has recently experienced a bottleneck in shifted mode, and this bottleneck may be due to threats from human habitat destruction. The consequences of this bottleneck and inbreeding depression could lead to the extinction of the CP population, necessitating genetic exchange for this population. In contrast, the probability of inbreeding depression was estimated to be low in the GP population, owing to the large population size.

### 4.2. Genetic Structure Populations

To the best of our knowledge, this is the first study to analyze the genetic structure of *M. longidorsalis* in the Hangang River system in South Korea to help establish conservation strategies. Interestingly, the CP populations showed less genetic mixing and some excess heterozygosity. For the CP population, this is the mid-stream point, and it can be assumed that there was little genetic flow between the other five populations at this point. One reason is that the points excluding the CP are the uppermost points of each water system, and the midstream point is only one CP point. Nevertheless, the populations, except for the CP site, were a mixture of the four clusters, which may be due to the high mobility of *M. longidorsalis*. However, this explanation does not explain the excess genotypes in the CP branch, and one of the other hypotheses could be that it is due to the small effective population size and altered genetic composition due to inbreeding.

Six *M. longidorsalis* populations had significantly low genetic differentiation in the microsatellite dataset. *P. tenuicorpa* inhabits the Hangang River water system, which is similar to the habitat of *M. longidorsalis*. The genetic structure of *P. tenuicorpa* is divided into the Namhangang and Bukhangang rivers [20]. In contrast, this study showed that the *M. longidorsalis* population was not divided into Namhangang and Bukhangang River genetic structures due to little genetic difference. This means that the K value in the STRUCTURE results cannot accurately divide the genetic structure, and the genetic structure is not clearly distinguished due to panmixia. A similar case based on similar STRUCTURE results has been reported previously [19]. STRUCTURE methods involve evaluating a predefined population and identifying new clusters. Preliminary incorrect specification can result in a waste of resources in habitat restoration by over-splitting populations that should be combined, thereby unnecessarily promoting gene flow [43,44]. Because DAPC is based on PC analysis, it can analyze genomic data sets relatively quickly and efficiently [45]. Additionally, the BIC value indicated the best fit cluster at 0 and the best fit at K = 1. The DAPC and BIC results showed that the scatter plot of individuals from each population consisted of a single population. Freshwater fish (*Coreoleuciscus splendidus* and *Coreoleuciscus aeruginos*) generally exhibit genetic differences across geographic water systems, with few genetic differences within the same system [40]. The *F*_ST_ results also showed low genetic differentiation, similar to *M. rapidus* (*F*_ST_: 0.001–0.014), suggesting that the six populations of *M. longidorsalis* were genetically one population [17].

The use of AMOVA to identify genetic variation between groups by grouping the Bukhangang River vs. Namhangang River and Imjingang River water systems showed very low differentiation (0.10%) in genetic variation between groups. In contrast, the within-group variation was 97.66%. This indicated that there was no genetic differentiation between the Bukhangang River and other rivers, strongly supporting them as a single group. Although their habitats are distributed in three areas (the Namhangang, Bukhangang, and Imjingang Rivers), their genetic structure suggests that they belong to one group. Therefore, even if any branch of the Namhangang, Bukhangang, or Imjingang Rivers becomes extinct, restoration is possible through artificial propagation if other branches survive. In other words, the group structure is highly vulnerable to extinction because of large-scale habitat destruction or pollution by human activities, as it is one population. This indicates an urgent need for the conservation of this species.

### 4.3. Conservation Implications

*M. longidorsalis* is an endemic species that only inhabits the Daedonggang and Hangang River water systems on the Korean Peninsula, which implies that if it becomes extinct in South Korea, it will be lost worldwide [15]. This is the first study to analyze the genetic diversity and structure of *M. longidorsalis* populations. We believe that our study will be helpful in conserving *M. longidorsalis* populations.

Typically, species management units are established by grouping genetically similar populations to avoid genetic disturbance [16]. The genetic structure of *M. longidorsalis* inhabiting the Daedonggang River could not be confirmed; however, *M. longidorsalis* inhabiting South Korea is a single population with a single genetic structure. The management unit for each species was determined based on its genetic structure. Therefore, *M. longidorsalis* should be managed as a single population in South Korea. *M. longidorsalis* populations require effective conservation strategies owing to their lower genetic diversity and significant inbreeding compared to *M. rapidus*. The sufficiently large GP population indicates that artificial propagation based on the GP population is necessary to increase and maintain CP population size.

However, inbreeding should be carefully considered when developing conservation strategies. The selective breeding of individuals that can maintain genetic diversity is necessary during restoration through artificial propagation regarding the inbreeding of *M. longidorsalis* in the Hangang River water system [42]. In addition, legally establishing conservation areas and preventing the construction of structures such as weirs and dams is necessary to avoid the destruction of the shallow habitat of *M. longidorsalis*. The population genetic analyses of *M. longidorsalis* conducted in this study will help conserve this species and establish future conservation strategies.

## Figures and Tables

**Table 1:** Genetic diversity based on 19 microsatellite loci in *M. longidorsalis*.

ID	Water System	N	*N*_A_	*H*_O_	*H*_E_	*P*_HWE_	*F*_IS_
JC	Namhangang River	20	8.47	0.625	0.741	0.000	0.132 ***
PC	Namhangang River	20	9.00	0.683	0.775	0.000	0.092 ***
GP	Bukhangang River	20	9.16	0.660	0.773	0.000	0.129 ***
HC	Bukhangang River	20	9.47	0.668	0.757	0.000	0.141 ***
CP	Bukhangang River	20	7.74	0.675	0.766	0.000	0.079 ***
PHC	Imjingang River	20	9.32	0.650	0.779	0.000	0.123 ***

N: number of samples; *N*_A_: number of alleles; *H*_O_: observed heterozygosity; *H*_E_: expected heterozygosity; PIC: polymorphic information content. *** *p* < 0.001.

**Table 2:** Summary statistics regarding bottleneck signature and effective population size estimated for populations at microsatellites.

Population ID	N	Wilcoxon Signed-Rank Test				N_e_	(95% CI)
		*P*_IAM_	*P*_TPM_	*P*_SMM_	Mode-Shift		
JC	20	0.000 ***	0.059	0.275	L-shaped	688	(101–∞)
PC	20	0.007 **	0.290	0.600	L-shaped	503	(99–∞)
GP	20	0.004 **	0.152	0.533	L-shaped	196	(75–∞)
HC	20	0.001 **	0.173	0.483	L-shaped	∞	(68–∞)
CP	20	0.000 ***	0.044 *	0.132	Shifted	78	(48–212)
PHC	20	0.000 ***	0.033 *	0.142	L-shaped	∞	(54–∞)

N: number of samples; N_e_: effective population size; *P*_IAM_: *p* value of bottleneck test using infinite allele mutation model; *P*_TPM_: *p* value of bottleneck test using two-phase mutation model (10% variance and 90% proportions of SMM); *P*_SMM_: *p* value of bottleneck test using stepwise mutation model; Ne: estimated effective population size using NeEstimator ver. 2.1 software; CI: confidence interval; * *p* < 0.05, ** *p* < 0.01, *** *p* < 0.001.

**Table 3:** Pairwise genetic differentiation of microsatellite (*F*_ST_) values among populations according to microsatellite analysis of *M. longidorsalis*.

	JC	PC	GP	HC	CP	PHC
JC	-	0.018	0.000	0.000	0.000	0.000
PC	0.013	-	0.054	0.000	0.000	0.054
GP	0.020	0.010	-	0.000	0.000	0.243
HC	0.034	0.015	0.019	-	0.000	0.153
CP	0.035	0.027	0.041	0.038	-	0.000
PHC	0.026	0.011	0.007	0.008	0.036	-

Pairwise genetic differentiation of significance level (above); pairwise genetic differentiation of microsatellites (below).

**Table 4:** Summary table of LnP (K) values using STRUCTURE HARVESTER (https://taylor0.biology.ucla.edu/structureHarvester/, accessed on 10 October 2023) website in *M. longidorsalis*.

K	Mean LnP (K)
1	−8928.47
2	−8930.61
3	−9034.84
4	−8931.72
5	−9091.46
6	−9240.74
7	−9546.24
8	−9198.53
9	−9453.05
10	−9585.59

**Table 5:** Summary information of the analysis of molecular variance for populations.

Source of Variation	d.f.	Sum of Squares	Variance Components	Percentage of Variance	*F*-Statistics
Microsatellite (two groups based on the water system (Namhangang River: JC, PC vs. Bukhangang and Imjingang Rivers: GP, HC, CP, PHC)					
Among groups	1	11.787	0.00618	0.10	0.023 ***
Among populations within groups	4	44.513	0.13287	2.23	0.022 ***
Within populations	234	1360.300	5.81325	97.66	0.001 *
Total	239	1416.600	5.95230	100.00	-

d.f.: degrees of freedom; * *p* < 0.05, *** *p* < 0.001; *F*-statistics are based on standard permutation across the full dataset.

## Data Availability

Microsatellite markers were deposited in GenBank (OR722786–OR722804).

## Supplementary Materials

The following supporting information can be downloaded at: https://www.mdpi.com/article/10.3390/genes15010069/s1, Table S1: Sample site information and number of *M. longidorsalis* individuals; Table S2: Summary microsatellite screening and raw data of long- and short-read sequencing in *M. longidorsalis*; Table S3: Nineteen microsatellite loci information developed from *M. longidorsalis*.

## References

[1] Frankham R. (1995). Conservation genetics. Annu. Rev. Genet..

[2] Buj I., Marčić Z., Flauder E., Šanda R., Vukić J. (2022). Population genetic structure of endemic fish species facilitating their survival in changing environments—A case study on the genus Telestes in Croatia. Diversity.

[3] Pimm S.L., Jenkins C.N., Abell R., Brooks T.M., Gittleman J.L., Joppa L.N., Raven P.H., Roberts C.M., Sexton J.O. (2014). The biodiversity of species and their rates of extinction, distribution, and protection. Science.

[4] Frankham R. (1998). Inbreeding and extinction: Island populations. Conserv. Biol..

[5] Furlan E., Stoklosa J., Griffiths J., Gust N., Ellis R., Huggins R.M., Weeks A.R. (2012). Small population size and extremely low levels of genetic diversity in island populations of the platypus, *Ornithorhynchus anatinus*. Ecol. Evol..

[6] Whitlock M.C. (2000). Fixation of new alleles and the extinction of small populations: Drift load, beneficial alleles, and sexual selection. Evolution.

[7] Frankham R., Ballou J., Briscoe D. (2010). Introduction to Conservation Genetics.

[8] Barnosky A.D., Matzke N., Tomiya S., Wogan G.O., Swartz B., Quental T.B., Marshall C., McGuire J.L., Lindsey E.L., Maguire K.C. (2011). Has the Earth’s sixth mass extinction already arrived?. Nature.

[9] Maxwell S.L., Fuller R.A., Brooks T.M., Watson J.E. (2016). Biodiversity: The ravages of guns, nets and bulldozers. Nature.

[10] Markert J.A., Champlin D.M., Gutjahr-Gobell R., Grear J.S., Kuhn A., McGreevy T.J., Roth A., Bagley M.J., Nacci D.E. (2010). Population genetic diversity and fitness in multiple environments. BMC Evol. Biol..

[11] Frankham R. (2005). Genetics and extinction. Biol. Conserv..

[12] Brauer C.J., Beheregaray L.B. (2020). Recent and rapid anthropogenic habitat fragmentation increases extinction risk for freshwater biodiversity. Evol. Appl..

[13] Gurevitch J., Padilla D.K. (2004). Are invasive species a major cause of extinctions?. Trends Ecol. Evol..

[14] Jeon H.B., Song H.Y., Suk H.Y., Bang I.C. (2022). Phylogeography of the Korean endemic *Coreoleuciscus* (Cypriniformes: Gobionidae): The genetic evidence of colonization through Eurasian continent to the Korean Peninsula during Late Plio-Pleistocene. Genes Genom..

[15] López-Pujol J., Martinell M.C., Massó S., Blanché C., Sáez L. (2013). The ‘paradigm of extremes’: Extremely low genetic diversity in an extremely narrow endemic species, *Coristospermum huteri* (Umbelliferae). Plant Syst. Evol..

[16] Kim I.S., Choi Y., Lee C.L., Lee Y.J., Kim B.J., Kim J.H. (2005). Illustrated Book of Korean Fishes.

[17] Hong Y.K., Sung H.C., Ko M.H., Kim K.S., Bang I.C. (2017). Distribution status and habitat characteristics of the endangered freshwater fish, *Microphysogobio rapidus* (Cyprinidae). Anim. Cells Syst..

[18] Palsbøll P.J., Berube M., Allendorf F.W. (2007). Identification of management units using population genetic data. Trends Ecol. Evol..

[19] Kim D.Y., Suk H.Y. (2020). Genetic diversity of the slender shinner (*Pseudopuntungia tenuicorpa*) and its conservational implications. Korean J. Ichthyol..

[20] Hong Y.K., Kim K.R., Kim K.S., Bang I.C. (2023). The impact of weir construction in Korea’s Nakdong River on the population genetic variability of the endangered fish species, rapid small gudgeon (*Microphysogobio rapidus*). Genes.

[21] Guo X.Z., Chen H.M., Wang A.B., Qian X.Q. (2022). Population genetic structure of the yellow catfish (*Pelteobagrus fulvidraco*) in China inferred from microsatellite analyses: Implications for fisheries management and breeding. J. World Aquacult. Soc..

[22] Kim K.R., Kwak Y.H., Sung M.S., Cho S.J., Bang I.C. (2023). Population structure and genetic diversity of the endangered fish black shinner *Pseudopungtungia nigra* (Cyprinidae) in Korea: A wild and restoration population. Sci. Rep..

[23] Wright J.M., Bentzen P. (1995). Microsatellites: Genetic markers for the future. Molecular Genetics in Fisheries.

[24] Hodel R.G., Segovia-Salcedo M.C., Landis J.B., Crowl A.A., Sun M., Liu X., Gitzendanner M.A., Douglas N.A., Germain-Aubrey C.C., Chen S. (2016). The report of my death was an exaggeration: A review for researchers using microsatellites in the 21st century. Appl. Plant Sci..

[25] Bolger A.M., Lohse M., Usadel B. (2014). Trimmomatic: A flexible trimmer for Illumina sequence data. Bioinformatics.

[26] Li H. (2013). Aligning Sequence Reads, Clone Sequences and Assembly Contigs with BWA-MEM. arXiv.

[27] Walker B.J., Abeel T., Shea T., Priest M., Abouelliel A., Sakthikumar S., Cuomo C.A., Zeng Q., Wortman J., Young S.K. (2014). Pilon: An integrated tool for comprehensive microbial variant detection and genome assembly improvement. PLoS ONE.

[28] Schuelke M. (2000). An economic method for the fluorescent labeling of PCR fragments. Nat. Biotechnol..

[29] Van Oosterhout C., Hutchinson W.F., Wills D.P.M., Shipley P. (2004). MICRO-CHECKER: Software for identifying and correcting genotyping errors in microsatellite data. Mol. Ecol. Notes.

[30] Kalinowski S.T., Taper M.L., Marshall T.C. (2007). Revising how the computer program CERVUS accommodates genotyping error increases success in paternity assignment. Mol. Ecol..

[31] Raymond M., Rousset F. (1995). Population genetics software for exact test and ecumenicism. J. Hered..

[32] Excoffier L., Lischer H.E. (2010). Arlequin suite ver 3.5: A new series of programs to perform population genetics analyses under Linux and Windows. Mol. Ecol. Resour..

[33] Piry S., Luikart G., Cornuet J.M. (1999). Computer note. BOTTLENECK: A computer program for detecting recent reductions in the effective size using allele frequency data. J. Hered..

[34] Maruyama T., Fuerst P.A. (1985). Population bottlenecks and nonequilibrium models in population genetics. II. Number of alleles in a small population that was formed by a recent bottleneck. Genetics.

[35] Cornuet J.M., Luikart G. (1996). Description and power analysis of two tests for detecting recent population bottlenecks from allele frequency data. Genetics.

[36] Luikart G., Cornuet J.M. (1998). Empirical evaluation of a test for identifying recently bottlenecked populations from allele frequency data. Conserv. Biol..

[37] Do C., Waples R.S., Peel D., Macbeth G.M., Tillett B.J., Ovenden J.R. (2014). NeEstimator v2: Re-implementation of software for the estimation of contemporary effective population size (Ne) from genetic data. Mol. Ecol. Resour..

[38] Pritchard J.K., Stephens M., Donnelly P. (2000). Inference of population structure using multilocus genotype data. Genetics.

[39] Evanno G., Regnaut S., Goudet J. (2005). Detecting the number of clusters of individuals using the software STRUCTURE: A simulation study. Mol. Ecol..

[40] Earl D.A., VonHoldt B.M. (2012). STRUCTURE HARVESTER: A website and program for visualizing STRUCTURE output and implementing the Evanno method. Conserv. Genet. Resour..

[41] Jombart T. (2008). Adegenet: A R package for the multivariate analysis of genetic markers. Bioinformatics.

[42] Kim K.R., Choi H.K., Lee T.W., Lee H.J., Yu J.N. (2023). Population structure and genetic diversity of the spotted sleeper *Odontobutis interrupta* (Odontobutidae), a fish endemic to Korea. Diversity.

[43] Weeks A.R., Sgro C.M., Young A.G., Frankham R., Mitchell N.J., Miller K.A., Byrne M., Coates D.J., Eldridge M.D., Sunnucks P. (2011). Assessing the benefits and risks of translocations in changing environments: A genetic perspective. Evol. Appl..

[44] Aitken S.N., Bemmels J.B. (2016). Time to get moving: Assisted gene flow of forest trees. Evol. Appl..

[45] Miller J.M., Cullingham C.I., Peery R.M. (2020). The influence of a priori grouping on inference of genetic clusters: Simulation study and literature review of the DAPC method. Heredity.

