# Supplementary material for: Assessment of the Genetic Diversity and Structure of the Korean Endemic Freshwater Fish Microphysogobio longidorsalis (Gobioninae) Using Microsatellite Markers: A First Glance from Population Genetics
Source: Genes (Basel). 2024 Jan 4;15(1):69. doi: 10.3390/genes15010069 (PMC10815670; doi:10.3390/genes15010069)
Supplement: Supplementary file 1 [file genes-15-00069-s001.zip › genes-2749107-supplementary.pdf]

Table S1. Sample sites information and number of individuals in the *M. longidorsalis*

Location	Site ID	Water system	N	Location
Jecheon stream	JC	Namhangang River	20	37°09'51"N 128°06'24"E
Hongjeongcheon stream	PC	Namhangang River	20	37°36'58"N 128°22'27"E
Gapyeongcheon stream	GP	Bukhangang River	20	37°49'48"N 127°31'03"E
Hongcheon River	HC	Bukhangang River	20	37°40'48"N 127°51'30"E
Jojongicheon stream	CP	Bukhangang River	20	37°43'56"N 127°25'15"E
Yeongpyeongcheon stream	PHC	Imjingang River	20	38°03'35"N 127°23'14"E

N: Number of samples.

Table S2. Summary microsatellite screening and raw data of long and short read sequencing in *M. longidorsalis*.

Raw data	Total reads	Total reads length (bp)
Long reads raw data	5,468,650	33,151,752,272
Short reads raw data	725,452,696	109,120,602,840
Microsatellite search		
Contig	6,220	
Total size of examined sequences (bp)	1,116,379,304	
Total number of identified SSRs	886,829	
Number of SSR containing sequences	5,004	
Number of sequences containing more than 1 SSR	4,898	
Number of SSRs present in compound formation	414,225	

Table S3. Microsatellite 19 loci information developed from *M. longidorsalis*

Locus	Primer sequence (5′→ 3′)	Motif repeat	Product size (bp)	Dye	N	N_A	H_O	H_E	PIC	GenBank Accession no.
Milo2	F: TGTAAAACGACGGCCAGTCCGAGAGCTGGATTCGACAA<br>R: TCCTGCTTCCAACCAAAGGT	(AC)17	158	FAM	27	15	0.741	0.899	0.873	OR722786
Milo9	F: TGTAAAACGACGGCCAGTTCGACACTGGGTTAAAAACTGT<br>R: GTGTTTTTCACCCAGTGTTGA	(ATA)9	188	FAM	26	14	0.654	0.878	0.846	OR722787
Milo11	F: TGTAAAACGACGGCCAGTGGGACTTCAATGGGAGGCAA<br>R: CAGCCCTGACTCATGAACGA	(AC)10	202	FAM	27	13	0.519	0.912	0.886	OR722788
Milo13	F: TGTAAAACGACGGCCAGTATCTCCGCACACATCGACTC<br>R: TTAGCGTCTCCTGGGAGTGA	(AC)12	208	FAM	27	7	0.815	0.776	0.726	OR722789

Milo17	F: TGTAAAACGACGGCCAGTCAGGGTTTCTGGGCTTGAGT<br>R: TAAATCCACAGCCCTGCACA	(AC)11	224	FAM	26	6	0.769	0.811	0.764	OR722790
Milo19	F: TGTAAAACGACGGCCAGTGCTCATCAGGCAGCAGGTAT<br>R: CAGGCCAACAGAAACACACG	(AC)10	237	FAM	27	10	0.889	0.846	0.811	OR722791
Milo20	F: TGTAAAACGACGGCCAGTCACCACTGTCTCCACTGTCC<br>R: CCGCGAACACACAACAAAGT	(CT)20	239	FAM	27	8	0.704	0.790	0.741	OR722792
Milo21	F: TGTAAAACGACGGCCAGTGGCACATTGCGCTCTCAATT<br>R: GGGGCTTTCAGGGATAGAGC	(CT)20	240	NED	27	22	0.704	0.959	0.938	OR722793
Milo25	F: TGTAAAACGACGGCCAGTCTGGATGTTCCTGTGGTGCT<br>R: ATGTGACTGCGGTCTTCCTC	(GT)17	255	NED	26	9	0.692	0.797	0.758	OR722794

Milo31	F: TGTAAAACGACGGCCAGTAAGGGGTTTAGGCACTTGGG<br>R: ATCACTGCTGAGGCTGGATG	(TTG)9	270	NED	26	16	0.692	0.877	0.848	OR722795
Milo42	F: TGTAAAACGACGGCCAGTGTGCCGAAACATCATGTGCA<br>R: AACTTGAGAGCGCCGTACAA	(TTTA)6	286	VIC	27	8	0.481	0.728	0.681	OR722796
Milo48	F: TGTAAAACGACGGCCAGTAGGTGTAGCCGCATTAGAGC<br>R: CATCAGACAAGCCCCAGTGT	(TGA)9	294	VIC	27	13	0.741	0.809	0.780	OR722797
Milo49	F: TGTAAAACGACGGCCAGTGTCCTGCACTGATTCCCCAA<br>R: TAGCAGGACCTCCACTAGCA	(AC)12	296	VIC	27	17	0.889	0.868	0.839	OR722798
Milo53	F: TGTAAAACGACGGCCAGTGTGGACCGGATAGCGTCAAT<br>R: GAATGCAGTTCAACCGGTCA	(TA)21	299	VIC	19	12	0.579	0.883	0.846	OR722799

Milo56	F: TGTAAAACGACGGCCAGTAGCAAGACCCTAGCAACCAC<br>R: TGAGTGGTTGCTACGCAGTT	(ATTG)6	309	VIC	26	8	0.654	0.662	0.624	OR722800
Milo61	F: TGTAAAACGACGGCCAGTAGCAGAGCACGTTACACACA<br>R: CCCTAAACCTCCACAGCGTT	(AC)10	323	VIC	26	3	0.154	0.277	0.253	OR722801
Milo63	F: TGTAAAACGACGGCCAGTAACATGGCGGACAACAAAGC<br>R: GAGGAGCTGGTTGAGAGTCG	(AGC)9	329	VIC	26	6	0.308	0.315	0.300	OR722802
Milo64	F: TGTAAAACGACGGCCAGTAATGATGCTCCCAGGCAACA<br>R: CATTCGGCTCCTCCTCCATC	(TGG)9	331	VIC	26	7	0.769	0.692	0.634	OR722803
Milo80	F: TGTAAAACGACGGCCAGTGGATAGTCTCACGCCTTCGG<br>R: TTGTGCAGTCCATCAACCCA	(AC)13	365	VIC	26	11	0.577	0.851	0.813	OR722804

N: Number of samples, N_A: Number of alleles, H_O: Observed heterozygosity, H_E: Expected heterozygosity, PIC: Polymorphic information content.
